# Cost and Cost-Effectiveness of Distributing HIV Self-Tests within Assisted Partner Services in Western Kenya

**DOI:** 10.3390/healthcare12191918

**Published:** 2024-09-25

**Authors:** Victor Mudhune, Monisha Sharma, Sarah Masyuko, Kenneth Ngure, George Otieno, Unmesha Roy Paladhi, David A. Katz, Edward Kariithi, Carey Farquhar, Rose Bosire

**Affiliations:** 1 Centre for Global Health Research, Kenya Medical Research Institute, Kisumu P.O. Box 1578-40100, Kenya; 2 School of Public Health, Jomo Kenyatta University of Agriculture and Technology, Nairobi P.O. Box 62000-00200, Kenya; 3 Department of Global Health, University of Washington, Seattle, WA 98105, USA; 4 PATH-Kenya, Kisumu P.O. Box 19128-40123, Kenya; 5 Department of Epidemiology, University of Washington, Seattle, WA 98105, USA; 6 Departments of Epidemiology and Medicine, University of Washington, Seattle, WA 98105, USA; 7 Centre for Clinical Research, Kenya Medical Research Institute, Nairobi P.O. Box 54840-00202, Kenya

**Keywords:** HIV testing, assisted partner services, HIV self-testing, costing, cost-effectiveness

## Abstract

**Background:** Assisted partner services (APS) is a recommended public health approach to promote HIV testing for sexual partners of individuals diagnosed with HIV. We evaluated the cost and cost-effectiveness of integrating oral HIV self-testing (HIVST) into existing APS programs. **Methods:** Within the APS-HIVST study conducted in western Kenya (2021–2022), we conducted micro-costing, time-and-motion, and provider surveys to determine incremental HIVST distribution cost (2022 USD). Using a decision tree model, we estimated the incremental cost per new diagnosis (ICND) for HIVST incorporated into APS, compared to APS with provider-delivered testing only. Scenario, parameter and probabilistic sensitivity analyses were conducted to explore influential assumptions. **Results:** The cost per HIVST distributed within APS was USD 8.97, largest component costs were testing supplies (38%) and personnel (30%). Under conditions of a facility-based testing uptake of <91%, or HIVST utilization rates of <27%, HIVST integration into APS is potentially cost-effective. At a willing-to-pay threshold of USD 1000, the net monetary benefit was sensitive to the effectiveness of HIVST in increasing testing rates, phone call rates, HIVST sensitivity, HIV prevalence, cost of HIVST, space allocation at facilities, and personnel time during facility-based testing. In a best-case scenario, the HIVST option was cheaper by USD 3037 and diagnosed 11 more cases (ICND = 265.82). **Conclusions:** Implementers and policy makers should ensure that HIVST programs are implemented under conditions that guarantee efficiency by focusing on facilities with low uptake for provider-delivered facility-based testing, while deliberately targeting HIVST utilization among the few likely to benefit from remote testing. Additional measures should focus on minimizing costs relating to personnel and testing supplies.

## 1. Introduction

Awareness of HIV status is critical for timely access to HIV treatment and prevention services. In Eastern and Southern Africa, the region most heavily impacted by HIV, testing rates have increased to 92% by 2022 [1]. As countries successfully scale-up HIV testing services (HTS), test positivity rates are declining [2], which has increased cost per new diagnosis [3]. There is a need for efficient HIV testing strategies that increase both reach (number of individuals newly tested) and yield (number of diagnoses per test performed).

In Kenya, an estimated 79.5% of people living with HIV (PLWH) are aware of their status [4]. Kenya adopted the World Health Organization (WHO) recommendation to implement assisted partner services (APS), a strategy to test the sexual and drug-injecting partners of PLWH [5], with potential for increased testing reach and yield. APS affordability has been shown to be highly sensitive to the uptake of testing services [6], hence diversifying options for partner testing, such as through community-based testing, provides an additional opportunity to increase program efficiency. Community-based HIV self-testing (HIVST) has been proposed to fill gaps in testing coverage and increase reach, especially among individuals unable to access existing HTS [7,8]. HIVST has been shown to be convenient, private, and highly acceptable among populations at risk of acquiring HIV [5]. HIVST and APS provide a possibility for program integration, whereby different kinds of services are combined to maximize efficiency and outcomes [9].

With reduced global financing for vertical HIV programs [10] and limited country budgets [11], the cost-effectiveness of current HIV testing strategies has become uncertain in low- and middle-income countries (LMIC). Targeted testing strategies that increase yield are more likely to be efficient [12,13]. Modeling analysis integrating available information on innovative HTS strategies can provide useful insights to efficiently increase yield under implementation uncertainties. Within a cluster randomized controlled trial evaluating the effectiveness of offering the option of oral HIVST versus provider-delivered testing to partners of APS index clients (APS-HIVST study) [14], we evaluated the incremental cost and cost-effectiveness of integrating HIVST into existing APS in public health facilities in Western Kenya. The costing was conducted and reported as per the reference case guidelines [15], while economic evaluation is reported in line with CHEERS 2022 guidelines [16].

## 2. Materials and Methods

### 2.1. Study Setting

We conducted this micro-costing within the APS-HIVST study [14]; a cluster randomized controlled trial conducted in 24 clinics in the Kisumu and Homa Bay counties of Western Kenya between March 2021 and June 2022. The trial’s objective was to determine the real-world effectiveness of offering oral HIVST (APS + HIVST) as an additional option for partner testing versus standard of care provider-administered testing alone (Standard APS) on the number of partners tested through APS. Study procedures are described elsewhere [14]. Briefly, participating healthcare facilities were largely government-run clinics and hospitals providing HIV care services supported by the Kenya Ministry of Health (MoH) and non-government organization (NGO) implementing partner (PATH Kenya). The facilities were selected based on geographical region, urbanicity, HIV testing volumes and APS performance; and balanced on the same characteristics across intervention and control arms. Individuals newly diagnosed with HIV were eligible for APS if they were ≥15 years of age, at low risk of intimate partner violence (IPV), not pregnant, and reported at least one sexual partner within the last 3 years, and able to provide partner contact information. HTS providers at participating facilities elicited partner contact information from consenting clients diagnosed with HIV (index clients). HTS counselors attempted to reach all named partners via phone and/or through in-person tracing. At APS + HIVST clinics, partners were notified of potential HIV exposure and offered HIV testing with the option to collect HIVST kits (OraQuick^®^) at designated community pharmacies or provider-delivered testing. For those choosing HIVST, HTS providers provided guidance via phone and made follow-up phone calls to partners to determine the test results. Those who reported a positive HIVST result were asked to confirm their test results through provider-delivered testing and subsequent linkage to care. As per local HIV testing guidelines, provider-delivered testing required serial tests with different rapid antibody test kits (Determine^®^ then First Response^®^) before a positive diagnosis [17], which was to be confirmed by a second HTS provider following the same testing algorithm. All HIV tests were provided for free.

### 2.2. Costing Approach

We estimated the incremental cost of HIVST integration into APS using a payer perspective, including staff time and resources needed to instruct identified partners on where to collect and how to use HIVST, follow-up for HIVST results, availing and dispensing HIVST at community pharmacies, and the costs of HIV testing in health facilities for those who test negative.

We conducted a health facility costing survey within the 12 APS + HIVST sites, determining fixed facility costs including utilities and administrative costs over one year period. In addition, we determined the floor space for HIV testing activities relative to other facility spaces, which was valued per square meter based on rental spaces for local commercial properties. We employed non-parametric bootstrapping with 1000 sampling rounds to obtain mean HTS area per test and average facility overhead allocation based on floor space per test across all facilities, with 95% confidence intervals. Supplies used for facility-based HIV testing were obtained via provider interviews, and summarized across facilities, adjusting for quantity of supplies used in quality assurance and repeat testing. We excluded supplies used by a second HTS provider in repeat testing for reactive tests as this was deemed common between the two arms in all scenarios. The unit cost of gloves, alcohol swabs, and sharps containers were obtained from Kenya Medical Supplies Agency (KEMSA) local price list [18], and the unit costs of test kits were obtained from Kenya government negotiated prices through the Global Fund, with inclusion of 25.1% import tax and 8% distribution fee as charged by KEMSA. HIV confirmatory tests using polymerase chain reaction (PCR) was excluded from this analysis, as the current national HTS guidelines require repeat testing after 14 days as opposed to molecular diagnosis in cases of discordant rapid diagnostic tests [17].

We conducted a fiscal survey with the implementing NGO. While this study was supported by an NGO, we excluded research and NGO specific costs, and estimated costs assuming implementation by the Kenya MoH. Government salaries were transformed into hourly wages based on assumption of 2081 h a year. The HTS providers completed a time-and-motion survey on activities conducted from notification of exposure to test results using HIVST, and for partners receiving provider-delivered HIV testing at the facility. From the time-and-motion surveys, we calculated the average amount of time spent on phone communication for both arms, with calling cost assigned based on call per minute rate as per the local service providers; we employed bootstrapping with 1000 samples to obtain the confidence intervals. Costs of physical tracing were excluded, as they are pre-test costs and assumed not to change based on the testing options. Training and vehicle costs were annuitized over a 5- and 10-year period, respectively, applying a 3% discount rate [15,19]. Supervisor and driver time, and vehicle costs were apportioned by work load as determined by participant volume at dispensing pharmacy relative to participant volume at health facilities. Annual training and pharmacy communication costs were considered fixed and divided by the number of individuals tested in the APS + HIVST arm. This study paid a fee to the pharmacies of KSh. 100 (USD 0.85) per dispensed HIVST, which was considered as fee for service delivery.

Downstream costs associated with post-HIV test procedures and antiretroviral therapy (ART) were excluded from the analysis. The incremental cost of integrating HIVST into APS is expressed in 2022 US dollars (USD), using a nominal exchange rate of KSh. 117.87 per USD [20]. The analysis was conducted using Microsoft Excel 365 (Microsoft Corporation, Redmond, WA, USA). The excel file detailing the costing methodology is included in the Supplementary Materials.

### 2.3. Outcome Data

We utilized effectiveness data from the APS-HIVST study [14], which found no significant differences between the two arms in HIV testing uptake among named partners of index clients (relative risk (RR): 1.02, 95%CI: 0.96–1.10) and new HIV diagnoses among named partners (adjusted RR: 1.03, 95%CI: 0.76–1.39). Based on APS-HIVST study population, the partners had 17% prevalence of undiagnosed HIV, uptake of facility-based testing at 96%, and a HIVST uptake rate of 83%. The confidence intervals for these parameters are shown in Table 1. The sensitivity and specificity of Determine^®^ and First Response^®^ were obtained from manufacturer’s specifications in documents submitted for respective WHO prequalification [21,22], while similar parameters for OraQuick^®^ were obtained from a field based evaluation within SSA [23].

### 2.4. Incremental Cost-Effectiveness

**Figure 1:**
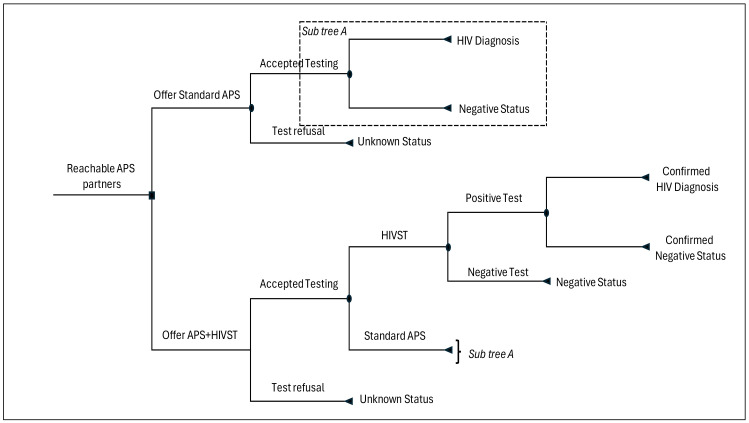
Decision tree model on HIV self-testing (HIVST) integration into assisted partner services (APS).

We developed a decision tree model (Figure 1) using TreeAge Pro 2023 R2.0 (TreeAge Software, Williamstown, MA, USA) to simulate provider incremental cost and outcomes when HIVST is integrated into an existing APS program within health facilities. The principal outcome was incremental cost per new HIV diagnosis in this setting. Given the effectiveness data in the APS-HIVST study did not demonstrate statistically significant differences between the options, this analysis focuses on identifying settings in which APS + HIVST would be cost-effective.

The model starts from a cohort of reachable individuals who have been identified as partners to index cases within APS. The provider’s decision point is to either offer provider-delivered testing only or offer HIVST at a community pharmacy as an additional testing option. Those offered HIVST may also opt for provider-delivered testing at the health facility or may decline testing altogether. The probabilities of these events were varied and included those obtained from APS-HIVST study data. Persons testing HIVST negative in the model are assumed to not require further evaluation, those who test positive are asked to come for confirmatory facility-based provider-administered testing. We assumed that all individuals who picked HIVST will use them, and all those with positive results will receive confirmatory testing. While it is possible to pick HIVST and not use it, the intensive follow-up in APS made such cases negligible, as those who did not want to pick up HIVST would likely opt for standard APS or refuse testing when offered. The model accounted for this under testing refusal, as this proportion was not explicitly captured within the APS-HIVST study. The probability of a positive HIVST is calculated as follows:

$${Prob}_{+ve}=\left\{\frac{Prev*{Sen}_{st}*S{en}_{t1}*{Sen}_{t2}}{\left(Prev*{Sen}_{st}\right)+\langle \left(1-Prev\right)\left(1-{Spec}_{st}\right)\rangle }\right\}+\left\{\left(1-\frac{Prev*{Sens}_{st}}{\left(Prev*{Sen}_{st}\right)+\langle \left(1-Prev\right)\left(1-{Spec}_{st}\right)\rangle }\right)\langle 1-{Spec}_{t1}*{Spec}_{t2}\rangle \right\}$$

where *Prev* denotes HIV prevalence in the APS population; *Sen* represents sensitivity of either HIVST (*Sen_st_*), Determine^®^ (*Sen_t_*_1_) or First Response^®^ (*Sen_t_*_2_); and Spec represents the specificity of either HIVST (*Spec_st_*), Determine^®^ (*Spec_t_*_1_) or First Response^®^ (*Spec_t_*_2_).

For those who test using provider-delivered testing, a serial testing approach using two blood-based antibody test kits was employed. We excluded costs of tie-breaker tests as this approach has been shown to achieve a positive predictive value of above the WHO 99% threshold [26], and frequencies of repeat tests would not vary significantly by study arm. The probability of a positive test outcome is calculated as follows:

$${Prob}_{+ve}=\left(Prev*{Sen}_{t1}*{Sen}_{t2}\right)+\left\{\left(1-Prev\right)\left(1-{Spec}_{t1}\right)(1-{Spec}_{t2})\right\}\;$$

The model inputs are presented in Table 1.

### 2.5. Sensitivity Analyses

We performed one-way sensitivity analyses for per minute phone call rate using minimum to maximum values based on local data adjusting for prices across SSA [27], HIV test kits were varied based on global market prices adjusting for global distribution costs [24,25], hourly HTS provider salary rate was varied based on provider qualification from certificate to degree applying local MoH pay scale, pharmacy HIVST dispensing fee was varied by ±50%, training was varied from no cost in a case where HIVST specific training is fully integrated into routine training to an increase by 50% above cost. Vehicle utilization cost varied from zero in a case of no vehicle use to 50% above cost, similarly, HIVST support personnel was varied from zero to 50% above cost. HIVST importation tax was varied from tax exempt to current tax rate, while rental space cost varied within price ranges for urban Kisumu and rural Homa Bay. HTS space allocation, communication cost, sensitivity and specificity of test kits were varied within their 95% confidence intervals. HIVST effectiveness data were identified through literature review relating to studies conducted in SSA countries to match the costing data. We restricted searches to studies published in the last 10 years to capture recent perception on HIVST and utilization. HIVST uptake rate [28], facility-based testing uptake rate [29], APS partner positivity rate [29,30] and effectiveness of HIVST in increasing testing [31,32] were incorporated into the one-way sensitivity analysis. We interpreted the resulting net monetary benefit (NMB) using a policy maker’s willingness-to-pay (WTP) threshold of USD 1000, based on correlation between incremental cost per new diagnosis and cost per DALY averted [3], using USD 1700 as the gross domestic product (GDP) per capita for SSA [33] assuming values below this threshold are very cost-effective.

In a scenario analysis, we evaluated cost-effectiveness in a modified-case scenario for HIVST based on combination of parameter estimates that minimize its cost and maximize its effectiveness. This scenario provides policy insight into maximum potential effect of HIVST integration when parameters that can be reasonably adjusted are in favor of HIVST in this setup. Costs relating to call rate, HIVST kit price, pharmacy dispensing fee, and pharmacy communication costs were set at the minimum limit, assuming a tax-exempt policy on HIVST kits. Assuming HIVST introduction was leveraging on resources from other services, costs relating to support personnel, vehicle utilization and startup training were removed. The sensitivity of HIVST was set to maximum depicting potential for blood based HIVST use, while HIVST utilization set to the minimum depicting a case of controlled use.

We performed probabilistic sensitivity analysis, varying all parameters within their base case uncertainty ranges with 10,000 permutations. Cost and rate variables assumed a gamma distribution, while probabilities assumed a beta distribution, where cost and rate would only vary based on scenarios, such parameters were set at prevailing conditions with ±50% variation (Table 1).

## 3. Results

The incremental cost of integrating HIVST into APS was estimated to be USD 8.97 per HIVST test conducted (Table 2). Cost drivers for HIVST integration were HTS supplies (USD 3.41, 38%) and personnel time (USD 2.65, 30%). While those testing positive using HIVST do not result in a cost saving to the health facility due to need for confirmatory provider-delivered facility-based testing, those who test negative using HIVST would avert a cost of USD 11.05 per test related to facility-based HIV testing. For this group, 65% of the foregone facility cost per test was resulting from personnel time (USD 7.21).

**Figure 2:**
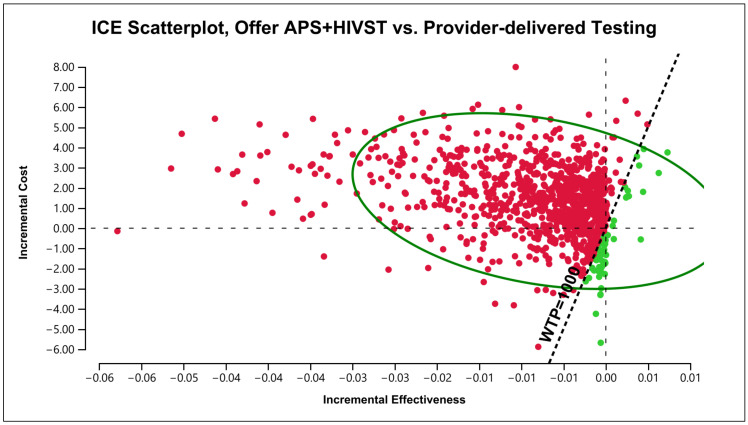
Incremental cost-effectiveness ratio planes under base-case analysis. APS—assisted partner services; HIVST—HIV self-testing; WTP—willingness to pay; ICE—incremental cost-effectiveness.

In the case of providing a HIVST option within APS, testing 6000 partners was associated with a USD 8500 higher cost and 37 fewer diagnosed cases compared to standard APS (ICER = −229.50). Figure 2 shows the incremental cost and effectiveness on the cost-effectiveness plane. The majority of the data points were in the north-western quadrant, indicating a higher cost and slightly lower effectiveness by adding HIVST into APS. However, a few data points were below the WTP threshold of USD 1000. The probability of the incremental cost-effectiveness ratio (ICER) being above the WTP threshold increased from 78% to 95% as WTP threshold increased to USD 1700, remaining at 95% for higher WTP values.

**Figure 3:**
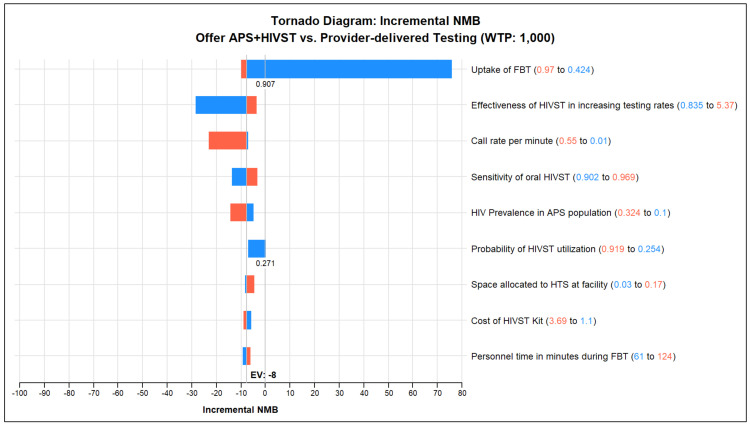
Tornado diagram of one-way sensitivity analysis on base case. NMB—net monetary benefit; WTP—willingness to pay; FBT—facility-based testing; HIVST—HIV self-testing.

The one-way sensitivity analyses at a policy maker’s WTP threshold of USD 1000 shows that the NMB appeared to be sensitive to the uptake of facility-based testing, the effectiveness of HIVST in increasing testing rates, phone call rates, HIVST sensitivity, HIV prevalence in the cohort, HIVST utilization rates, cost of HIVST, space allocation for HTS at facilities, and personnel time during facility-based testing (Figure 3). Results were largely robust to all remaining parameters.

**Figure 4:**
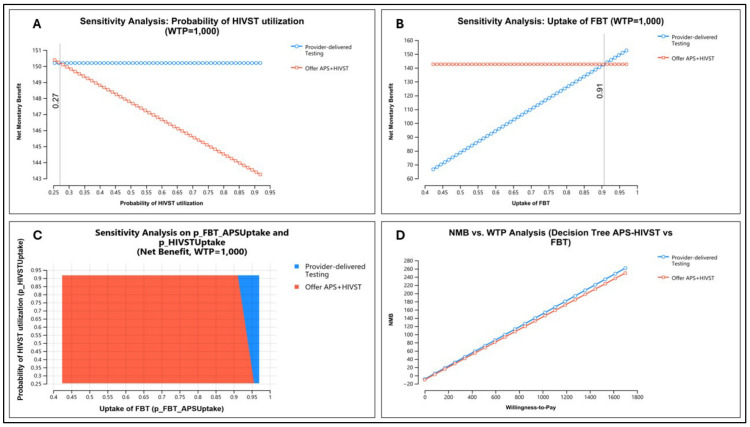
One-way and two-way sensitivity analysis on net monetary benefit (NMB). (**A**) One-way sensitivity analysis of HIVST utilization rates at WTP = USD 1000. (**B**) One-way sensitivity analysis of FBT uptake rates at WTP = USD 1000. (**C**) Two-way sensitivity analysis of FBT uptake on HIVST utilization rates at WTP = USD 1000. (**D**) One-way sensitivity analysis of WTP threshold. NMB—net monetary benefit; HIVST—HIV self-testing; APS—assisted partner services; FBT—facility-based testing.

Our base case threshold analysis indicates that adding HIVST to APS may be cost-effective at a facility-based testing uptake of less than 91% and HIVST utilization rates of less than 27% (Figure 4). Two-way sensitivity analysis shows that for facility-based testing uptake less than 90%, HIVST introduction is supported across the range of HIVST utilization rates. The overall decision did not change based on WTP values when varied from zero to USD 1700 (GDP per capita for SSA in 2022).

In a modified-case scenario, where some factors could be manipulated in this setting to favor HIVST implementation, standard APS was dominated as APS + HIVST was cheaper by USD 3037 and diagnosed 11 more cases (ICER = 265.82).

## 4. Discussion

We estimated the incremental cost and cost-effectiveness of offering HIVST within APS compared to provider-delivered facility-based testing only, and identified conditions under which such implementation may be more efficient. For individuals who test positive, HIVST introduced additional costs to APS, while for those who test negative, there was a relative cost saving due to reduced personnel utilization at the health facility. Under conditions of low facility-based testing uptake and HIVST utilization rates, it is possible for HIVST integration into APS to be cost-effective, hence implementers and policy makers should ensure that such programs are implemented under conditions that guarantee efficiency in diagnosing new cases.

The incremental cost of HIVST integration into APS was estimated as USD 8.97 per test, while those testing negative and do not have to go to the health facility for confirmatory testing, resulted in a potential cost saving of USD 11.05 per test. Different from full implementation cost, the incremental cost is the additional cost of providing HIVST as a testing option on top of standard APS provision. HIVST integration needs may be underestimated if the APS program is not running efficiently. Systematic review of HIVST economic studies show significant disparity in cost estimates, largely resulting from differences in costing methods [34], and HIVST implementation approaches [35,36], which further highlight factors that can affect efficient HIVST utilization. Our findings are similar to other studies evaluating the incremental cost of introducing HIVST to ongoing HTS. The incremental cost of integrating HIVST into public health facilities in SSA ranged from USD 4.27 to USD 13.42 per kit distributed [37], while a study conducted across countries in West Africa, found HIVST integration ranged from USD 4 to USD 26 per kit distributed [36]. In this analysis, while the HIVST option was associated with higher costs mainly attributed to testing supplies, there was potential for cost saving at facility level largely attributed to differences in personnel time. Consistent findings were noted within the ATLAS project conducted across three countries in West Africa [36], where HIVST cost was largely driven by cost of personnel (27–68%) and test kits (32–73%), with similar findings in HIVST facility integration studies across countries in Southern Africa [37,38]. Task-shifting to lower trained HTS provider cadres and negotiating for lower purchase and distribution cost per HIVST kits are likely to significantly reduce the overall program cost.

We found that within APS programs where the uptake of facility-based testing is high (>91%), offering HIVST as a testing option is unlikely to be cost-effective unless HIVST utilization was low (<27%). HIVST has been widely documented as more costly relative to facility-based testing [39], partly due to requirement for repeat testing for positive test outcomes as confirmatory test at the health facilities. To be cost-effective, HIVST introduction needs to be in settings with lower testing coverage [40], subsequently improving testing uptake and increasing number of new diagnoses, as opposed to simply offering alternative testing options to those already utilizing available HIV testing services. While a higher cost has been documented previously [38,41], fewer outcomes resulted from HIVST being incorporated into a serial testing algorithm, with the advantage of increasing the positive predictive value but with potential for fewer confirmed positive cases [26]. This algorithm comes with downstream benefits attributed to fewer false positives, hence cost saving from averted unnecessary antiretroviral initiation, which has been shown to be a greater cost compared to retesting [42]. There have been concerns regarding lower sensitivity of HIVST relative to blood-based tests [43], potentially contributing to the difference in outcome observed. False-negative cases are likely to propel transmission, with implications for individual and public health as well as health systems costs. However, over an extended period, repeat testing may mitigate effects of any false negatives attributed to lower sensitivity of HIVST. The WHO recommends the three-test algorithm in regions with HIV prevalence <5%, and the two-test algorithm for regions with a prevalence >5% [44]. In this high-prevalence population, a potential approach to reduce implementation cost would be to simplify a facility-based testing algorithm following a positive test outcome with HIVST; however, more research is needed to understand concerns and gains related to this approach.

We present a decision analysis model to guide implementation in a setup where HIV prevalence is relatively high, with high uptake rates for both facility-based testing and HIVST. The sensitivity analyses emphasize areas to reduce HIVST implementation costs, including need for cheaper approaches to contacting clients, need to minimize program vehicle utilization, downward negotiation of HIVST kit prices including potential tax exemption, leveraging MoH activities to support HIVST introduction through integrated training, support personnel and potential of dispensing at community outreaches. While other studies have shown that positivity rate is a key determinant of cost-effectiveness [45]; APS partners have a relatively high positivity rate hence this did not feature as a key variable. Our modified-case scenario shows that HIVST has potential to be more efficient than facility-based testing, with lower costs and more new diagnoses. Programs will need to effect measures that ensure lower costs and deploy strategies for HIVST that guarantee reach to specific population subgroups facing barriers to facility-based testing. We utilized costing from a payer perspective likely to leave out societal benefits relating to testing at home as opposed to the facility, where partners and HTS providers may save time and cost related to traveling. Saved time may result in gains from alternative activities. We assumed high linkage to care and confirmatory testing, which may not reflect all settings. Improved models could evaluate cost-effectiveness from societal perspective with varying linkage to care.

The strengths of this study include using data from a randomized controlled trial and published literature, use of bottom-up micro-costing, and extensive sensitivity analysis with parameters from different settings. However, our study had several limitations. First, we used a decision tree model with a limited period for related costs and benefits. Economic evaluation models projecting related data over a lifetime or a longer time period are more accurate in guiding decision making [46]. Second, we used self-reported time-and-motion surveys necessitated by the inability of data collectors to be present real-time during procedures for the HIVST arm as they were largely remote. Limitations of self-reported time-and-motion surveys result from differences between respondents on what constitutes an activity [47], as indirect time accounts for a significant proportion of the daily working hours of a provider. The pre-designed time-and-motion instrument may have reduced this bias, but not eliminated it. Third, our outcome of interest was reported as cost per diagnosis, instead of cost per quality adjusted life years (QALYs) or disability adjusted life years (DALYs), hence may be more difficult to compare with other public health interventions. However, cost per diagnosis has been shown to be strongly correlated with cost per DALY averted for HTS [3], and can be used as a metric to assess an intervention’s cost-effectiveness.

## 5. Conclusions

As combined intervention, APS and HIVST the have potential to increase testing yield by promoting overall testing uptake. HIVST utilization remains promising in APS situations where partners face barriers in accessing facility-based provider-delivered testing. In situations or population segments with high uptake for facility-based testing, HIVST integration into APS is unlikely to be cost-effective, hence its utilization should be carefully considered and limited to population segments likely to test based on its availability. Additional measures to ensure HIVST integration is cost-effective should focus on task-shifting for HTS support personnel, and policies to reduce the cost of HIVST to match those of blood-based HIV tests.

## Figures and Tables

**Table 1:** Model input parameters in base case.

Description	Distribution	Standard Deviation		Base Case (Range)	Ref.
**Costs**					
Call rate per minute	Gamma	0.01		0.03 (0.02–0.04)	
Vehicle utilization cost	Gamma	0.16		0.63 (0.32–0.95)	
HTS supplies for +ve test outcome	Gamma	0.07		1.41 (1.36–1.63)	
HTS supplies with −ve test outcome	Gamma	0.14		3.07 (2.75–3.29)	
Facility overhead allocation	Gamma	0.03		0.07 (0.03–0.13)	
Cost of HIVST kit	Gamma	0.65		2.6 (1.1–3.69)	[24,25]
Tax applicable to HIVST kits ^a^	Fixed	-		21.5%	
Cost of distribution ^a^	Fixed	-		8%	
Cost of HTS provider per hour	Gamma	0.43		3.62 (2.95–4.65)	
Dispensing fee for HIVST	Gamma	0.21		0.85 (0.42–1.27)	
Cost of rental space (per annum/m^2^)	Gamma	10.68		28.61 (8.55–51.28)	
Pharmacy communication costs	Gamma	0.02		0.11 (0.06–0.17)	
Cost of initial training on HIVST	Gamma	0.02		0.07 (0.035–0.105)	
Cost of HIVST support personnel ^b^	Gamma	0.07		0.29 (0.15–0.44)	
		**Alpha**	**Beta**		
**Probabilities**					
HIV Prevalence in APS population	Beta	589	2944	0.17 (0.15–0.19)	[14]
Probability of APS uptake in FBT	Beta	1422	67	0.96 (0.94–0.97)	[14]
Sensitivity of Determine RDT	Beta	399	1	1.00 (0.991–1.00)	[21]
Specificity for Determine RDT	Beta	593	7	0.9893 (0.978–0.996)	
Sensitivity of First Response RDT	Beta	399	1	1.00 (0.992–1.00)	[22]
Specificity for First Response RDT	Beta	598	2	0.997 (0.995–1.00)	
Sensitivity of HIVST	Beta	376	24	0.941 (0.902–0.969)	[23]
Specificity of HIVST	Beta	598	2	0.997 (0.993–0.999)	
Probability of HIVST utilization	Beta	2111	46	0.83 (0.81–0.84)	[14]
		**Standard deviation**			
**Utilization rates**					
Personnel time during FBT (mins)	Gamma	16.22		93 (61–124)	
FBT partner phone tracing (mins)	Gamma	3.07		27 (22–34)	
HTS space allocated (m^2^/test)	Gamma	0.02		0.06 (0.02–0.11)	
HIVST phone communication (mins)	Gamma	3.51		31 (24–38)	
Effectiveness of HIVST in increasing testing rates	Normal	0.035		1.02 (0.96–1.10)	[14]
HIVST personnel time (mins)	Gamma	5.90		44 (33–57)	

HTS—HIV testing services; HIVST—HIV self-testing; FBT—facility-based testing; RDT—rapid diagnostic test. ^a^ Based on local applicable cost as per country program. ^b^ Includes apportioned coordinator and driver time.

**Table 2:** Incremental cost and foregone facility-based costs related to introducing HIVST into APS.

**INCREMENTAL COST PER HIVST TEST**					
**Fixed Costs**	**Measure**	**Units**	**Total Cost**	**Unit Cost**	**Percent**
***Capital Items***					
Vehicle	Annuitized and apportioned, per test	3897	USD 2457	USD 0.63	7%
***Support Activities***					
Support Personnel	Apportioned, per unit	3897	USD 1128	USD 0.29	3%
Pharmacy Communication	Annual, per unit	3897	USD 428	USD 0.11	1%
Startup HIVST Training	Annuitized, per unit	3897	USD 282	USD 0.07	1%
	Total			USD 1.10	12%
***Variable Costs***	**Measure**	**Units**	**Unit Cost**	**Total Cost**	
Personnel	Minutes per follow-up	44	USD 0.06	USD 2.65	30%
Communication	Minutes per follow-up	31	USD 0.03	USD 0.96	11%
HTS Supplies ^a^	per test	1	USD 3.41	USD 3.41	38%
Pharmacy Dispensing Fee	per test	1	USD 0.85	USD 0.85	9%
	Total			USD 7.87	88%
**TOTAL COST**				**USD 8.97**	100%
**FOREGONE COST OF FACILITY-BASED HIV TEST (HIVST NEGATIVE)**					
**Activity**	**Measure**	**Units**	**Unit Cost**	**Total Cost**	**Percent**
***Fixed Costs***					
Av. Overhead Allocation	Apportioned per test	1	USD 0.38	USD 0.38	3%
Av. HTS Space Allocation	Apportioned per test	0.04	USD 28.61	USD 1.21	11%
***Variable Costs***					
Communication	minutes per follow-up	27	USD 0.03	USD 0.84	8%
Personnel	Minutes per test + follow-up	120	USD 0.06	USD 7.21	65%
HTS Supplies ^b^	per test	1	USD 1.41	USD 1.41	13%
**TOTAL COST**				**USD 11.05**	100%

HTS—HIV testing services; HIVST—HIV self-testing; APS—assisted partner services. ^a^ Includes cost of HIVST Ex works, international import costs, applicable local taxes and local distribution cost. ^b^ Includes cost of Determine^®^, gloves, alcohol swabs and sharps container (apportioned).

## Data Availability

The original contributions presented in this study are included in this article/Supplementary Materials, further inquiries can be directed to the corresponding author.

## Supplementary Materials

Spreadsheets used for cost analysis can be downloaded at https://zenodo.org/records/13371061 (accessed on 13 September 2024).
